# Supplementary material for: Large local variations in the use of health services in rural southern Ethiopia: An ecological study
Source: PLOS Glob Public Health. 2022 May 25;2(5):e0000087. doi: 10.1371/journal.pgph.0000087 (PMC10021478; doi:10.1371/journal.pgph.0000087)
Supplement: S2 Table — (DOCX) [file pgph.0000087.s002.docx]

**S2 Table:** **The annual health service utilization rate of new cases in kebeles of Dale and Wonsho districts, 2017/18, Sidama, Ethiopia.**

| **Kebele Name** | **Kebeles with health centre** | **District Name** | **Annual visits per each kebele** | **Population size per kebele** | **Annual utilization rate per person** |
| --- | --- | --- | --- | --- | --- |
| Semen Mesenkela | Yes | Dale | 1493 | 8958 | 0.17 (0.16 0.18) |
| Gidamo | Yes | Dale | 809 | 8672 | 0.09 (0.09 0.1) |
| Dagiya | Yes | Dale | 2212 | 10647 | 0.21 (0.20 0.22) |
| Moto | Yes | Dale | 1806 | 10909 | 0.17 (0.16 0.17) |
| Semen Kege | Yes | Dale | 3264 | 7390 | 0.44 (0.43 0.46) |
| Wene Nata | Yes | Dale | 908 | 3984 | 0.23 (0.21 0.24) |
| Goyida | Yes | Dale | 1383 | 8991 | 0.15 (0.15 0.16) |
| Bera Chale | Yes | Dale | 2508 | 10877 | 0.23 (0.22 0.24) |
| Megera | Yes | Dale | 1497 | 8146 | 0.18 (0.17 0.19) |
| Bua Bedagelo | Yes | Dale | 2996 | 8548 | 0.35 (0.34 0.36) |
| Bokaso town | Yes | Wonsho | 3491 | 2944 | 1.19 (1.15 1.23) |
| Andenya Fero | Yes | Wonsho | 2110 | 8588 | 0.25 (0.24 0.26) |
| Gudumo | Yes | Wonsho | 1659 | 8226 | 0.20 (0.19 0.21) |
| Gajaba | Yes | Wonsho | 1472 | 9706 | 0.15 (0.14 0.16) |
| Hunkute | Yes | Wonsho | 3127 | 9839 | 0.32 (0.31 0.33) |
| Danshe Sire | No | Dale | 744 | 8798 | 0.08 (0.08 0.09) |
| Soyama | No | Dale | 691 | 10128 | 0.07 (0.06 0.07) |
| Chume | No | Dale | 1383 | 8447 | 0.16 (0.16 0.17) |
| Wayicho | No | Dale | 725 | 9154 | 0.08 (0.07 0.09) |
| Degara | No | Dale | 1140 | 13560 | 0.08 (0.08 0.09) |
| Duba | No | Dale | 770 | 13254 | 0.06 (0.05 0.06) |
| Halile | No | Dale | 803 | 8595 | 0.09 (0.09 0.10) |
| Shoye | No | Dale | 608 | 7271 | 0.08 (0.08 0.09) |
| Gane | No | Dale | 644 | 3394 | 0.19 (0.18 0.20) |
| Ajawa | No | Dale | 1270 | 6891 | 0.18 (0.17 0.19) |

| **Kebele Name** | **Kebeles with health centre** | **District Name** | **Annual visits per each kebele** | **Population size per kebele** | **Annual utilization rate per person** |
| --- | --- | --- | --- | --- | --- |
| Debub Mesankela | No | Dale | 901 | 7151 | 0.13 (0.12 0.13) |
| Shifa | No | Dale | 317 | 4631 | 0.07 (0.06 0.08) |
| Debub Kege | No | Dale | 308 | 4981 | 0.06 (0.06 0.07) |
| Tula | No | Dale | 193 | 5037 | 0.04 (0.03 0.04) |
| Hedha Kaliti | No | Dale | 370 | 4344 | 0.09 (0.08 0.09) |
| Kaliti Simita | No | Dale | 1109 | 7396 | 0.15 (0.14 0.16) |
| Awada | No | Dale | 31 | 3536 | 0.01(0.006 0.01) |
| Masincho | No | Dale | 34 | 5359 | 0.01 (0.004 0.01) |
| BeraTedicho | No | Dale | 1829 | 9050 | 0.20 (0.19 0.21) |
| Wara | No | Dale | 1109 | 10606 | 0.10 (0.10 0.11) |
| Wicho | No | Dale | 1635 | 8632 | 0.15 (0.15 0.16) |
| Gajamo | No | Dale | 1395 | 9701 | 0.14 (0.14 0.15) |
| Shefina | No | Dale | 1381 | 10428 | 0.13 (0.13 0.14) |
| Lelo | No | Dale | 1428 | 6731 | 0.21 (0.20 0.22) |
| Hayilo | No | Dale | 1331 | 8494 | 0.16 (0.15 0.17) |
| Manche | No | Dale | 1546 | 8879 | 0.17 (0.17 0.18) |
| Halekena | No | Wonsho | 726 | 8697 | 0.08 (0.08 0.09) |
| Menafesha | No | Wonsho | 850 | 9949 | 0.09 (0.08 0.09) |
| Kinante | No | Wonsho | 625 | 6744 | 0.09 (0.09 0.10) |
| Barabalcho | No | Wonsho | 760 | 7125 | 0.11 (0.10 0.11) |
| Homo | No | Wonsho | 396 | 3746 | 0.11 (0.10 0.12) |
| Orowo | No | Wonsho | 593 | 3108 | 0.19 (0.18 0.21) |
| Gishire | No | Wonsho | 255 | 4564 | 0.06 (0.05 0.06) |
| Lalamo | No | Wonsho | 1377 | 7119 | 0.19 (0.18 0.20) |
| Bokaso rural | No | Wonsho | 2321 | 6997 | 0.33 (0.32 0.35) |
| Huletenya Fero | No | Wonsho | 1518 | 10241 | 0.15 (0.14 0.16) |
| Kiliye | No | Wonsho | 1262 | 7699 | 0.16 (0.16 0.17) |
| Meniho | No | Wonsho | 1177 | 6771 | 0.17 (0.16 0.18) |
| Mamena | No | Wonsho | 1388 | 7726 | 0.18 (0.17 0.19) |
| Total |  |  | 67678 | 421359 |  |

**S2 Table continued**
