# Supplementary material for: Large local variations in the use of health services in rural southern Ethiopia: An ecological study
Source: PLOS Glob Public Health. 2022 May 25;2(5):e0000087. doi: 10.1371/journal.pgph.0000087 (PMC10021478; doi:10.1371/journal.pgph.0000087)
Supplement: S3 Table — (DOCX) [file pgph.0000087.s003.docx]

**S3 Table: Most likely and secondary spatial clusters of low health service utilisation detected after adjustment of covariates in purely spatial analysis in Dale and Wonsho districts, Sidama, southern Ethiopia, 2017/18.**

| **Cluster** | **Number of cluster locations** | **Likelihood ratio** | **P_value** |
| --- | --- | --- | --- |
| Most likely cluster | 23 | 3834.32 | 0.001 |
| Secondary cluster | 2 | 466.10 | 0.001 |
| Secondary cluster | 1 | 186.45 | 0.001 |
| Secondary cluster | 1 | 24.62 | 0.001 |
| Secondary cluster | 1 | 12.78 | 0.001 |
| Secondary cluster | 1 | 10.86 | 0.002 |
