# Supplementary material for: Large local variations in the use of health services in rural southern Ethiopia: An ecological study
Source: PLOS Glob Public Health. 2022 May 25;2(5):e0000087. doi: 10.1371/journal.pgph.0000087 (PMC10021478; doi:10.1371/journal.pgph.0000087)
Supplement: S4 Table — We did the analysis using aggregated data from 54 kebles (n = 54 kebeles), R-squared = 0.15, P-value = 0.04. (DOCX) [file pgph.0000087.s004.docx]

**S4 Table: Multivariable linear regression analysis of geographic factors affecting the health service utilisation in Dale and Wonsho districts, Sidama, southern Ethiopia, 2017/18.**

| **Geographic variables** | **Beta** | **Standard error** | **P-value** | **95% CI** | **Variance inflation factor** |
| --- | --- | --- | --- | --- | --- |
| Attitude (Elevation) | 0.10 | 0.07 | 0.12 | -0.03, 0.25 | 1.24 |
| Population density | 0.05 | 0.07 | 0.43 | -0.09, 0.20 | 1.21 |
| Distance from the health centre | -0.05 | 0.02 | 0.02 | -0.08, -0.01 | 1.32 |

We did the analysis using aggregated data from 54 kebles (n=54 kebeles), R-squared=0.15, P-value=0.04
