# Supplementary figures and images for: Large local variations in the use of health services in rural southern Ethiopia: An ecological study
Source: PLOS Glob Public Health. 2022 May 25;2(5):e0000087. doi: 10.1371/journal.pgph.0000087 (PMC10021478; doi:10.1371/journal.pgph.0000087)

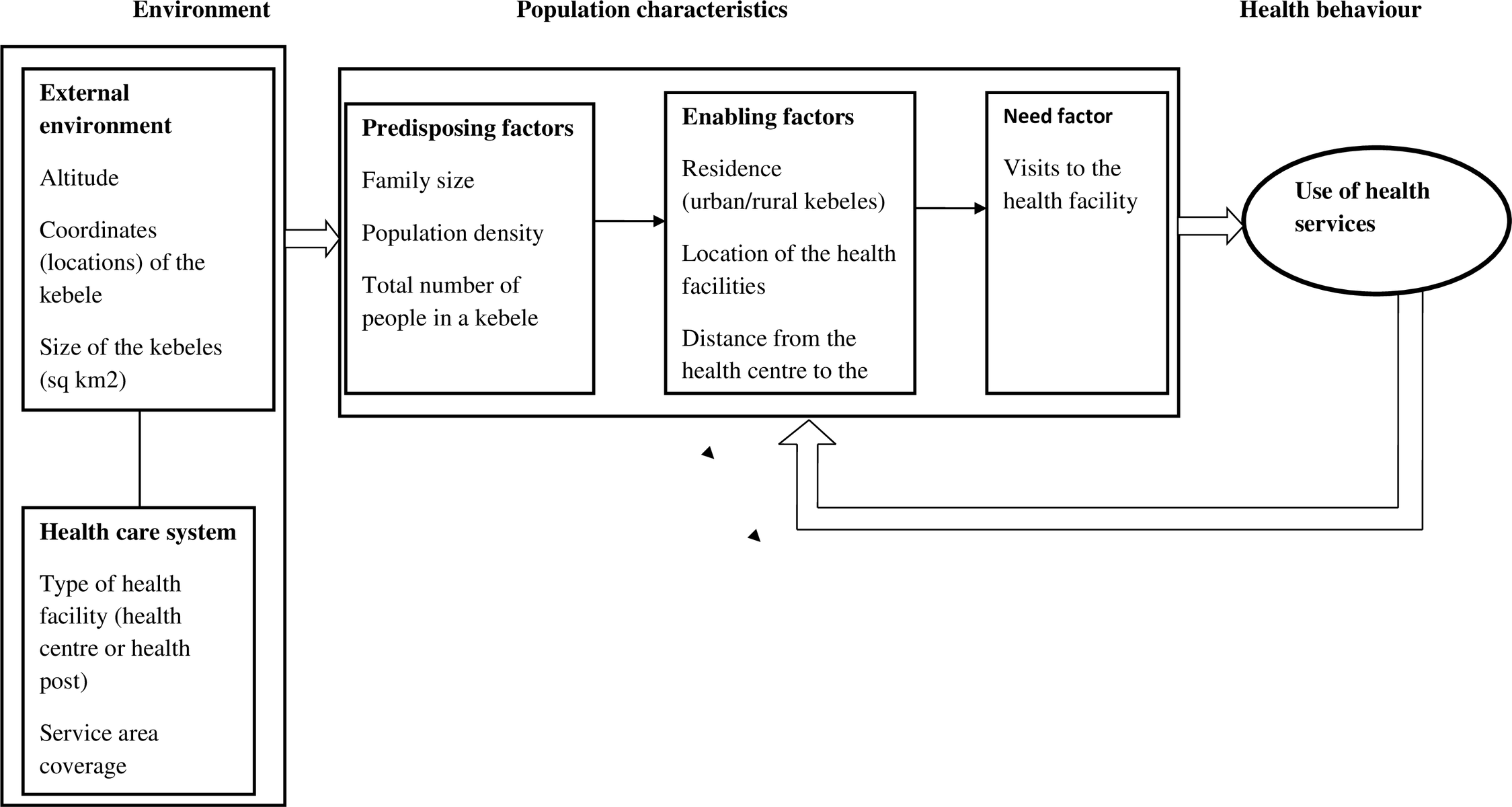

Supplement: S1 Fig — (TIF) [file pgph.0000087.s005.tif]

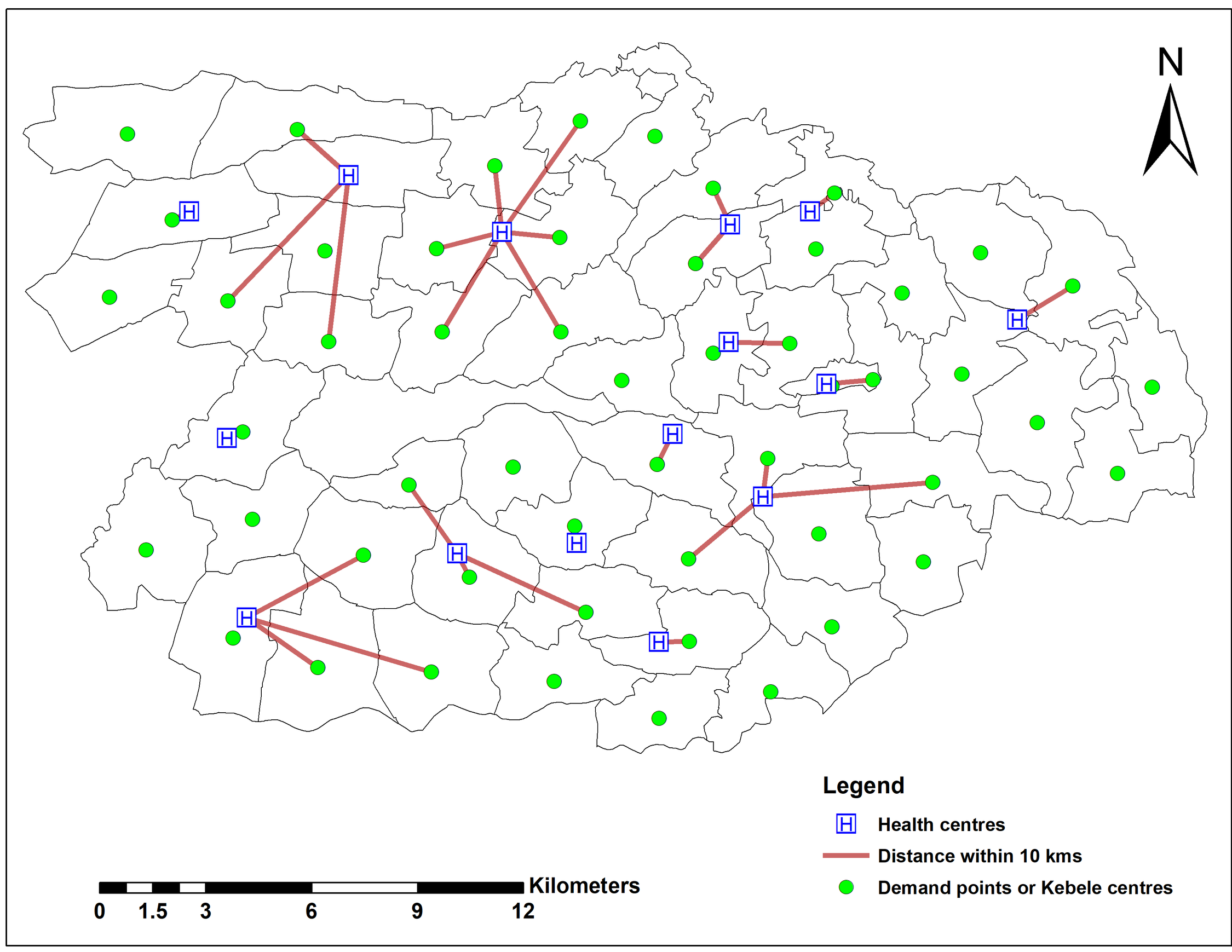

Supplement: S2 Fig — (TIF) [file pgph.0000087.s006.tif]
