# Supplementary material for: Large local variations in the use of health services in rural southern Ethiopia: An ecological study
Source: PLOS Glob Public Health. 2022 May 25;2(5):e0000087. doi: 10.1371/journal.pgph.0000087 (PMC10021478; doi:10.1371/journal.pgph.0000087)
Supplement: Attachment — Submitted filename: Response to Reviewers_03_11_2021.doc [file pgph.0000087.s007.doc]

**Editor’s comment**

Journal Requirements:

1. Please provide us with a direct link to the base layer of the map used in 2-5 and ensure this location is also included in the figure legend.

**Response:** Dear Editor, for this study we have used the coordinates from the Central Statistical Agency of Ethiopia. The coordinates were exported to ArcGIS to create the base layer of the map. These maps are in line with other previous studies done in the study area. For the health centre locations we used Google Earth map. However, we have now changed the points by replacing it with local GPS data (*Pease see the revised methods section, page 10-11, line 213-221*).

*“Geographical information for the study kebeles was collected from different sources. Geographically weighted central locations were indicated by the kebele central locations as coordinates. We extracted the geographical coordinates (latitudes and longitudes) of the kebeles from a previous study done in the region collected by using the geographic positioning system (GPS) tools [35]. These coordinates were used to build the base-maps of the study area and point-maps of the health centres. The Central Statistical Agency of Ethiopia collects geographical data and distributes to the regions. Therefore, population density, family size and altitude information were obtained from the Central Statistical Agency, Hawassa branch, and Sidama Region Plan Commission.”*

**Reviewer #1**

**Comment:** Abstract. The author should state explicitly that Secondary data was used. They should state the number of Kebeles studied. The variation of the coverage or visits/ population / perpetuation and an overall mean or median.

**Response:** Thank you for pointing out the issues. We have included the information in the abstract and in the results now. The detail of the visits per population data can also be found in the previous study done by the same authors (Areru HA, Dangisso MH, Lindtjørn B. Low and unequal use of outpatient health services in public primary health care facilities in southern Ethiopia: a facility-based cross-sectional study. BMC Heal Serv Res 2021 211. 2021 Aug;21(1):1–14.) ( (*Please see the revised abstract section, page 2, line 28-31, 35-36; result section, page 14, line 293-294)*.*“An exploratory ecological study design was employed on the secondary patient data collected from 1 July 2017 to 30 June 2018 from 65 primary health care units of the fifty-four kebeles in Dale and Wonsho districts, in the Sidama region.”*

*“The distribution of the health service utilisation varied across the kebeles with a mean of 0.17 visits per person per year (Range: 0.01-1.19).”*

*“The mean annual health service utilisation rate was 0.17 visits per person per year (S2 Table).”*

**Comment:** Introduction. There is the need to rearrange some part of this. The area dealing with use of geographic analysis should come only after the main problems of coverage has been put perspective. Reference 10 is rather 30years old. The research question should be more pointed and the justification. Much of the part informing readers of Ethiopia’s plans to improve its health system may not be useful and not relevant.

**Response**: We have re-arranged the introduction based on your suggestion now. We have removed and modified some parts of the Ethiopian plan to improve the health system. However, we kept some important information because it will support our findings and conclusions on improving accessibility and ultimately service usage. The health extension program is the country’s main mechanism of achieving universal health coverage. We also added the research question and some more justification of the study (*Please see the revised introduction section, page 5, line 87-97, 98-103; page 6, line 112-115*).

*“The upgraded health facilities will be equipped with better health professionals, clinical services, infrastructure, governance and leadership, and information systems [15–17]. In this new plan, health posts that are far (>1 hour) from health centres, and in geographically inaccessible areas, will be upgraded to “comprehensive health posts”. In addition to the basic health extension packages, the comprehensive health posts will provide improved clinical and curative services. During this 15 years plan (2020-2035), a health centre and health post will be merged if they are close to each other. These improvements could increase the coverage, accessibility, quality and service usage [17].”*

We used reference 10, because there was no study that assessed the geographical variation of health service utilization by including health posts, health centres and hospitals in multiple administrative levels (villages, towns, woreda or awraja, and region). Most studies conducted currently fail to show the geographical variation of over-all health service utilization, or were conducted at community level and focused on specific health services.

*“Specific questions we seek to investigate are: (1) How is health service utilisation distributed at kebele level? (2) What kinds of kebele level geographic factors affect the service usage? The answer to these questions might give direction to policy-makers on improving the health service accessibility.”*

**Comment:** Methods were sound but it should be stated that Secondary data were used and that the study sites were purposive chosen.

**Response:** Thank you for your suggestion. We have added and clarified the type of data we used in the methods section now (*Please see the revised methods section, page 8-9, line 171- 173; page 10, line 200-203*).

*“We selected the districts purposively since they were the study sites for the previous demographic study done by the same authors [33].”*

*“Secondary data was collected from the standard registers supplied by the Federal Ministry of Health to the health centres and health posts. Each unit or department had a registry with the registration, identification and service related information. The information reviewed from the registries is attached in S1 Table.”*

.
**Comment:** Results. Line 266, the RR values for the most likely cluster should be cross checked.

**Response:** Thank you for pointing out the mistake. We have made the correction now (*Please see the revised results section, Table 1, page 16, line 324-326).*

**Comment:** Discussion. Line 306. The authors should show why gave think that there are no variations between household legal and kebele level. The implications of the fi dings have not been discussed adequately

**Response:** We accepted your suggestion. We have elaborated the points in the limitations section of the discussion. The exposure variables we used in this study were altitude, population density, family size and distance from the health centre to the centre of the kebele. We excluded family size from the analysis because there was no difference in the average number of people per household in the kebeles. These variables are less likely to vary within small geographic areas, such as kebele. Therefore, we can say that the ecological fallacy that results from translating the group level finding to the individual level is negligible in our case. We have added some explanations as you suggested (*Please see the revised discussion section, page 18-19, line 368-371).*

*“Besides, most of the exposure variables included in this study, altitude, population density, and distance from the health centre to the centre of the kebele, were unlikely to be different at kebele and individual levels. This implies that the findings observed at kebele level holds true at individual level as well.”*

**Reviewer #2**

**Comment:** Despite this, the manuscript will require revision to clarify many issues, beginning with the health access conceptual framework and definitions for coverage and access. The authors must provide background information to understand the context, including demographic composition, epidemiological profile, and more detail about the health care facilities resources and registry system. The discussion section requires to be connected with the health care access conceptual framework.

**Response:** Thank you very much for your deep analysis and evaluation of our paper. We have addressed all the points raised in the specific comments section below.

**Specific comments:**
**Comment**: **Line 55: Please define coverage and access. It requires a definition of coverage, access, and utilization.**

**Response:** Thank you very much for the insight. We have added the definition of coverage, access and health service utilization. We also described the Anderson’s conceptual framework adopted to include all the determinants of health service use *(Please see the revised introduction section, page 3-4, line 57-69; page 6, line 110-112, Supplementary figure 1:* Conceptual framework based on the behavioural model of health service use by Ronald M. Andersen, 1995*).*

*“Primary health coverage is defined as access to primary health care (health centres and health posts) in relation to the total population [6]. Service access consists availability, affordability, and acceptability, accommodation, affordability and acceptability dimensions [7,8]. Health care utilization is the description or quantification of health care service usage [9]. R. Andersen proposed a behavioural health service use model with major components that influence the health outcome. Population factors were considered as major predictors for health service use and health outcomes. These factors include predisposing (demographic, health beliefs, values, attitude, and knowledge), enabling (availability of health service, income, travelling and waiting time) and need factors. The other determinants in the model were the health care system and external environmental factors. Health service use was considered as an outcome on previous version of the model, but it is considered as a determinant of subsequent health outcome in the latest version [10,11].”*

*“We also integrated the determining factors in the context of the behavioural model of health service use by Ronald M. Andersen (S1 Fig.).”*

**Comment**: **Line 57: Clarify that this is a national definition said afterward, but this is the first mention.**

**Response:** We have made the correction by stating that the definition is from the national guideline *(Please see the revised introduction section, page 4, line 72-73).*

*“According to the national criteria, within 2 hours of walking distance or less than 10 km away from the primary health care unit are considered physically accessible [6].*

**Comment: Line 65: Explain the differences between health posts and health centers.**

**Response:** We appreciate your suggestion. We have explained the difference between the health post and health centre based on the Ethiopian three-tire health service delivery system (*Please see the revised introduction section, page 4, line 82-86).*

*“According to the Ethiopian three-tire health service delivery system, one health centre serves 25,000 rural population up to 40,000 urban population. There are five community health posts under each health centre. Each health posts serve 3,000 -50000 population Five health posts and one referral health centre comprise a primary health care unit [14,15].”*

**Comment**: **Line 103 Please add a little detail about additional health determinants such as % women, % of children, poverty. And if it is possible leading causes of death and morbidity**.

**Response:** Thank you for your suggestion. We a have managed to include the demographic composition of women and children. We also could find the leading causes of morbidity in under-five years’ age group. Yet, there is a limited published literature for the leading cause mortality and morbidity in adults in the study region. We have added a literature with a cohort study from southern-central Ethiopia (Butajira) health and demographic site from 13 years ago to indicate the perceived causes of mortality in similar settings (*Please see the revised methods section, page 7, line 136-140).*

*“Females compose 49.5 % of the population. Under-five years of age children constitute 15.8% of the population [25]. Based on the regional reports, pneumonia was considered the most common cause of morbidity in under-five children [23]. An earlier study from southern-central Ethiopia showed malaria as a major perceived cause of death among adults [26].”*

**Comment**: **Line 132: Provide more detailed pieces of information about the selected centers' registries and their integrity or quality. Specify the way the authors acceded to those registries.** **Provide the rationale to include only new cases; this should be related to the definition of health care utilization in the conceptual framework**.

**Response:** All the registers were standardized formats distributed by the Federal Ministry of Health to all health centres and health posts throughout the country. Every registry has information about the registration date and card number, visit type or date, patient’s name and demographic information, specific service expected to be delivered or diagnoses, and remark or appointment. We obtained permission from the region’s and districts’ health offices. By showing the permission letter, we were allowed to review the registries. This didn’t create a problem on the routine work processes because we were using archived registries from the previous year (*Please see the revised methods section, page 10, line* 200-203; and line 205-208).

*“Secondary data was collected from the standard registers supplied by the Federal Ministry of Health to the health centres and health posts. Each unit or department had a registry with the registration, identification, and service related information. The information reviewed from the registries is attached in S1 Table.”*

*“The data collectors went to the service delivery units with permission letters from each district and copied the information into the data collection format from the respective registries in the study period.”*

However, a recent study showed some quality problem in the routine health management information system in Ethiopia (*Adane A, Adege TM, Ahmed MM, Anteneh HA, Ayalew ES, Berhanu D, et al. Routine health management information system data in Ethiopia: consistency, trends, and challenges. Glob Health Action. 2021;14 (1)*). Our data might not be free from these quality problems because it is extracted from similar routine health management information system. However, the quality problem was present in every type of health facility and registries. Therefore, the bias resulting from this might cancel out each other because we didn’t leave any functional primary health care unit from both districts during data collection and analysis (*Please see the revised discussion section, page 18, line* 362-365).

*“Moreover, the quality of data from the health service information system might be poor [40]. However, since we used all registries from all health centres and health posts from Dale and Wonsho districts, there might not be non-differential information bias.”*

On the other hand, while collecting the data we couldn’t be sure of the number of times the repeated service users came to the health facility. In addition, the national health management information guide states, the numerator for ten most common causes of morbidity should include only new cases. The health service utilisation rate also used new visitors as a numerator. Therefore, we included only new cases in the analysis (*Please see the revised methods section, page 8, line 169-171; page 9, line 191-192*).

*“We used all new cases or patients who sought health services from all health centres and health posts in the Dale and Wonsho districts. The national health management information guide indicates to use new cases as a numerator for morbidity calculations [12].”*

*“Health service utilisation rate is the number of new visits to health facilities per year relative to the total population of the same geographical area.”*

**Comment**: **Line 141 Explain the utilization of the 1.5 design effect.**

**Response:** Thank you for raising the issue. We did the sampling calculation to determine the sample size needed. The sample size obtained from the calculation based on the estimates (14,246) was below what we used for the spatial analysis from the census of the health facilities (67,678). Thus, we can be sure that our study has adequate power and minimum margin of error.

**Comment**: **Line 152: Give more detail of the data collection instrument to the health care registries system and how the authors acceded them.**

**Response:** We have added the information about the registries and information included in the data collection format as a supplementary file now. We accessed the registry from each service unit after we explained to the health centers’ and health posts’ managers about the study and showed the permission letter we obtained from the regional and districts health office authorities (official letter was given to us) (*Please see the revised methods section, page 10, line 200-203, Supplementary table 1: List of registers reviewed to extract the data from health centres and health posts in Dale and Wonsho districts; page 10, line 205-208*).

*“Secondary data was collected from the standard registers supplied by the Federal Ministry of Health to the health centres and health posts. Each unit or department had a registry with the registration, identification, and service related information. The information reviewed from the registries is attached in S1 Table.”*

*“The data collectors went to the service delivery units with permission letters from each district and copied the information into the data collection format from the respective registries in the study period.”*

**Comment: Line 161: Clarify the operational definition used to measure the variable distance to health centers. It seems that is a proxy.**

**Response:** We accepted your suggestion to put operational variable in the methods section. Distance to health centres was defined in our study as the distance measured in kilometres from the health centre in the kebele to the centre of the kebele. Because we did not have individual household data, we considered the centre of the kebeles as a proxy indicator of accessibility. We have included this definition and other operational definitions of variables in the methods section now (*Please see the revised methods section, page 9-10, line 190-196*).

***“Operational definitions***

*Health service utilisation rate is the number of new visits to health facilities per year relative to the total population of the same geographical area.*

*Family size is the average number of people living together in a household in a kebele.*

*Distance from the health centre to the centre of the kebele is the distance measured in kilometres from the health centre in the kebele to the centre of the kebele. It is a proxy indicator for access* [12]*.”*

**Comment**: **Line 166: It seems that the authors used the distance to health centers and not to health posts, clarify.**

**Response:** This is a correct observation. Our unit of analysis was at kebele level. There could be more than one health posts in each kebele. Using distance from health post locations might be used if our unit of analysis was at household level. The health posts were not located to be accessible from each corner of the kebeles, rather they are nearby to their community. Moreover, we considered the mean distance from the health facilities to be approximated to the kebele centres based on a previous study done in the area (*Dangisso MH, Datiko DG, Lindtjørn B. Accessibility to tuberculosis control services and tuberculosis programme performance in southern Ethiopia. Glob Health Action.2015;8(1)*) (*Please see the revised methods section, page 11, line 222-225*).

*“The distance information was generated by the ArcGIS 10.3 (Esri Inc., Redlands, CA, USA, 2014) analysis tool by proximity-near command from the kebele central location and health centre location files. We used distance from health centres to the centre of the kebele because our unit of analysis was at kebele level not household level.”*

**Comment: Line 189: Define service area coverage.**

**Response:** Service area coverage is a type of network analysis in ARC GIS. Service area coverage is the area within a given distance or travel time or any impendence unit from the nearby health facility. We have presented the definition in the methods now (*Please see the revised methods section, page 12, line 247-249)*.

*“Service area coverage is defined in this study as the area measured through physical distance between the service area (health centres) and the people benefiting from the service (kebele centre)* [37,38].”

**Reviewer #3**

**Comment:** This is a very well written and clear manuscript of an ecological study assessing the variation in health service utilisation in rural southern Ethiopia. I spotted a couple of minor typos (line 191 missing the word 'was' used to ... and line 317 4th word 'that' should be removed). I would like further detail on what constitutes 'family size', this is an unfamiliar term to me (perhaps an example added to the text would suffice). Otherwise the analysis method is well considered and explained, and the conclusions well supported and justified. An enjoyable read, I hope this work has impact to support improvement in health service accessibility.

**Response:** Thank you very much for your constructive reviews. We have removed all typing and grammatical errors as you suggested. We also included the definition of family size used in this study in the operational definition section (*Please see the method section, page 9, line 193*)

*“Family size refers to the average number of people living together in a household in a kebele.”*
